# Formation and Schema Analysis of Oil Painting Style Based on Texture and Color Texture Features under Few Shot

**DOI:** 10.1155/2022/4125833

**Published:** 2022-06-13

**Authors:** Yuanyuan Zhao

**Affiliations:** Guangxi Normal University for Nationalities, Chongzuo 532200, China

## Abstract

Texture has strong expressiveness in picture art, and color texture features play an important role in composition. Together with texture, they can convey the artistic connotation of portrait, especially in oil painting. Therefore, in order to make the picture form oil painting style and oil painting schema, we need to study the texture and color texture in combination with the previous oil painting art images. But now, there are few samples of good oil paintings, so it is difficult to study the texture and color texture in oil paintings. Therefore, in order to form a unique artistic style of modern oil painting and promote the development of modern oil painting art, this paper studies the texture and color texture characteristics in the environment of few oil painting works. This paper establishes a model through deep neural network to extract the image incentive and color texture of oil painting art works, which provides guidance for promoting the development of oil painting art. The experiments in this paper show that the depth neural network has high definition for the extraction of texture and color texture of small sample oil painting images, which can reach more than 85%. It has high guiding significance for the research and creation of oil painting art.

## 1. Introduction

For artistic creation, the first thing is to be based on the shoulders of predecessors. Therefore, it is very necessary to study the works created by predecessors, but it is also a difficult problem to fully study the previous works of art, especially the development of oil painting art. From the perspective of iconography, the texture that can best reflect the artistic language in oil painting art can deeply convey the painter's creative thought and emotion [1]. The second is the color texture, which can reflect the beauty of oil paintings, especially the combination of color can give people a visual impact. But at present, there are not many excellent oil painting works left by predecessors. In order to carefully extract the incentive and color texture in the image in a few works and play its role in oil painting art creation, it is necessary to establish the recognition model of image texture and color texture in a small sample environment, study the internal structure and formation elements of texture and color texture, and promote the formation and schema analysis of oil painting style in modern art.

In this paper, the extraction and analysis of texture and color texture features in the image can not only promote the development and creation of modern art, but also promote the development of modern art education. It makes the development of art more rapid but also has internal meaning. Furthermore, the study of texture and color texture features can help to integrate modern and traditional oil painting art, transforming modern painting into an oil painting style. It is more vibrant and creative. The importance of exploring texture in contemporary oil painting is enormous. The texture composition can reflect the creator's style and spirit while also increasing the aesthetic value of the work. From previous people's oil paintings, the logical relationship between texture, color, and texture features is examined. In their respective systems, they create styles. It can aid students in their exploration and creation of oil painting art. It allows people who practise painting to self-diagnose problems with their work and improve their aesthetic ability after viewing the article.

In order to make modern art images from oil painting style, many people have studied the texture, color, and texture of images. Liu's image oil painting style transfer reconstruction algorithm based on generative countermeasure network was difficult to train. He proposed an improved generative countermeasure network based on gradient punishment and constructed the total variance loss function to study the migration and reconstruction of image oil painting style. The experimental results show that the algorithm has better performance of image oil painting style migration and reconstruction and better effect of image oil painting style migration and reconstruction [2]. However, his research does not specifically explain how the oil painting style is transferred. Yang had proposed a joint domain image stylization method for portrait oil painting. From the perspective of art appreciation, a large number of oil painting works have been analyzed. Three key factors are summarized: texture, structure, and other image. Based on this, a sample-based color adjustment method is proposed. A large number of experimental results show that this method has achieved good results in maintaining the consistency of given image content [3]. His research only studied the color of the image, and the research has a certain one sidedness. El Mehdi et al. proposed a fast and efficient image indexing and search system based on color and texture features in content-based image retrieval system. The experimental results show that the combination of color features and texture features of brightness components can obtain similar results, and the efficiency is higher [4]. His research lacks experimental data and cannot reflect the accuracy of his conclusions. Do et al. proposed a CBIR system based on semantic features to retrieve images from databases. Their method divides the query image into 100 regions and uses K-means clustering algorithm and KNN classification algorithm for semiautomatic annotation. The experimental results showed that the image retrieval efficiency of the system is more than 98% [5]. His research has no specific theoretical support and is not convincing. In order to improve the efficiency of oil painting creation, the pyramid reference image sequence was established by using bilateral reference filtering and mathematical morphology operations by Chen. The experimental results show that the new reference image sequence has clearer texture direction, clear boundary, and is easy to draw. At the same time, this method can be well applied to oil painting teaching [6]. His research has studied the texture features of images, but the construction process of pyramid reference image sequence is not very systematic. Based on the research of these people, this paper reconstructs the recognition model of texture and color texture features in the image under a few samples so as to promote the formation of image oil painting style and graphic analysis.

In this paper, the research on the formation of oil painting style and graphic analysis has the following innovations: (1) this paper applies deep neural network to how to analyze the texture and color texture features in the image, and constructs a model system based on neural network, which can recognize more accurate texture and color texture features in the case of few samples. (2) In this paper, the texture and color texture of the image are analyzed with the help of neural network model, and then the image schema is analyzed with the algorithm. It enables the oil painting style to be transmitted and promotes the formation of oil painting style. (3) In this paper, a series of experiments are carried out on the research of color texture features. It proves that the model of this paper can promote the development of art biography and the formation of oil painting style.

## 2. Methods of Oil Painting Style Formation and Schema

### 2.1. Texture, Color, and Texture Characteristics of Oil Painting

**Figure 1:**
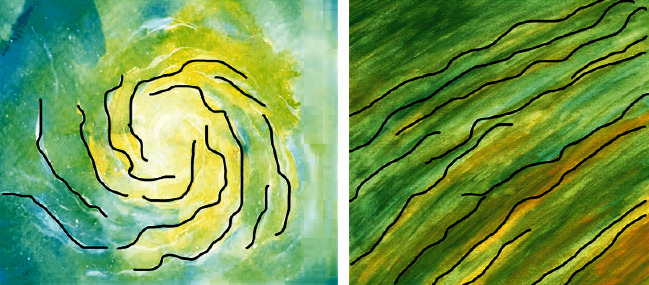
Oil painting texture.

No matter how a painting is handled, it will produce brush texture, so texture is one of the important formal elements of constructing portraits. With its rich artistic expression, it plays an important role in the field of painting and exists in oil painting in a unique form. Through the expression form of its own unique attributes, it shows the aesthetic feeling of vision and form in oil paintings [7]. The texture of different materials can show the characteristics of artistic language and image on the picture. In oil painting texture, texture can be said to be a kind of texture. Texture refers to the aesthetic feeling caused by the real expression of texture in the image of plastic arts. It is not only the texture of oil painting materials themselves but also the formation of new image texture after integration with texture materials. It is the combination of oil painting materials and texture materials [8]. In addition, the combination of texture materials and optimized materials will inevitably produce new different visual effects, so it will make people who look at images form different visual or tactile feelings. Texture characteristics refer to the special properties of all actual object surfaces, such as clouds, trees, bricks, hair, and buildings. People are used to some local disordered features in the image, but the whole has regular features, which are also called textures. It also contains important information about the arrangement of surface structure and its relationship with the environment. Texture is also a very important but difficult feature in the image, as shown in Figure 1.

**Figure 2:**
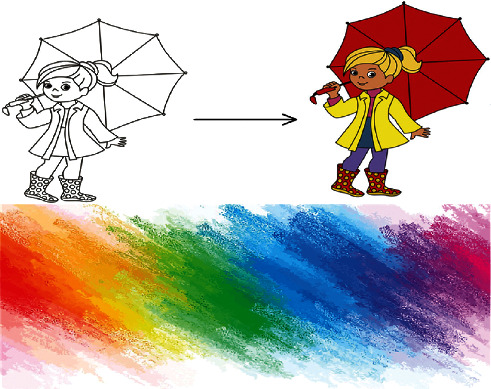
Image color.

In Figure 1, it can be seen that the Figure on the left is visually different from the Figure on the right. The Figure on the left is visually a water vortex image. The picture on the right is a water grain image, which has different visual effects. And from the perspective of feeling, the picture on the left will be more fresher, while the picture on the right will be heavier. In terms of feeling, in addition to the different effects of texture, we can also see the direction of the painter from the texture. It shows the traces left intentionally and unintentionally in the process of painting. These painting traces can obviously feel the emotional changes in the process of painter's creation. The rich and strong collocation of colors will make people form a completely different vision. Therefore, the color feature is not only the most direct and prominent feature of the image but also one of the most important sensory features of graphic vision [9, 10], as shown in Figure 2.

The contrast before and after colouring can be seen clearly in Figure 2. The portrait in the picture does not match the real human image when it is not painted, but after painting, it can see the skin color and hair color clearly and has a better sense of hierarchy. As a result, texture can reflect the painter's emotional changes, and texture and color features can provide people with visual effects. At the same time, the painter's thoughts and feelings will be conveyed through color intensity. Its bright, warm color expresses the painter's inner love. Texture composition is how you express texture. Its concave, thick, and light surface will evoke a variety of emotions in those who view it. The introduction and creation of texture effects in painting art have opened up a whole new world for the expression of form and color. It has a natural, fresh, and flexible appearance, as well as the ability to be skillfully borrowed. It has evolved into a one-of-a-kind language for artists to express their emotions. It makes natural beauty and subjective feelings blend harmoniously in artistic creation [11]. Therefore, in order to make a breakthrough in modern art creation, we need to analyze the texture and color texture of previous art works.

### 2.2. Oil Painting Style Formation and Schema

Schema is to serve the formal beauty in painting. Schemata can be divided from time, regional environment, cultural background, personal artistic language, and so on. In all paintings, personal artistic language is to combine the things to be painted in the picture according to their feelings, and strengthen their feelings by using the basic rules of composition [12]. Painting is to match dots, lines, faces, black, white, gray, color blocks, etc. It uses the feeling to mix the elements such as points, lines, and surfaces with scattered, size, rhythm, straightness, cold, and warm. The art of painting must fully understand a pair of contradictions such as virtual reality, square and round, black and white, density, rhythm and charm, and experience the aesthetic principles contained here.

**Figure 3:**
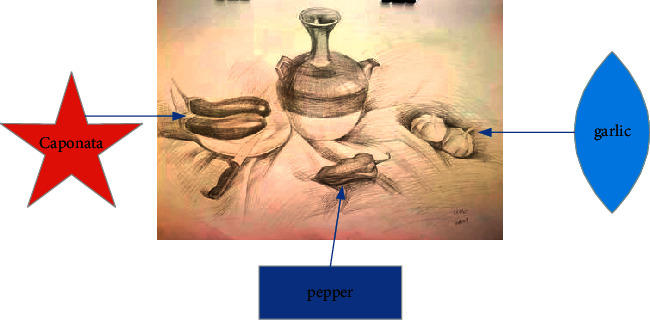
Placement of composition and shape.

The three-dimensional characteristics [13] of natural objects should be transformed into two-dimensional through both eyes, and the painter's composition ability is fully reflected in this sensitivity. The position of the object image in the picture and the size proportion of the object image will have different visual and psychological feelings. How to form the characteristics of the image in the composition on the basis of clarifying the modeling system and color system becomes very important [14]. There is also an important portrait skill in the image, that is, the placement of portrait modeling. Modeling is how the painter deals with the placement of objects in the painting. Modeling system refers to a unique way formed by the painter's unique treatment of various factors constituting the picture. Modeling is not a single visual grasp, simple object reproduction. Graphic appearance writing is the active pursuit of perceptual psychology and an activity of the interaction between subject consciousness and object spiritual essence [15]. The establishment and improvement of the modeling system is a sign of the maturity of a painter. The modeling system does not take whether the picture has an image as the judgment basis of whether there is a modeling system. Conceptualizing the image is by no means the establishment of a modeling system. Because the painter's quality and taste determine his aesthetic ability and artistic appreciation. The establishment of modeling system requires painters to have high quality and taste, and they can also take this as a breakthrough point to form their own artistic style. The arrangement of composition and shape is shown in Figure 3.

The oil painting style is both traditional and contemporary. We must first understand the oil painting style in order to form it in the transmission of modern art. Oil painting styles and genres are diverse, and there have been countless styles since its inception [16]. Every painter will absorb the essence of the classic paradigm in order to create something new. It defies convention by employing open reading and its own oil painting creation style, discussing new artistic connotations and expression forms, and attempting to establish its own independent painting style. As a result, when creating modern art, we must consider the style of classic oil paintings. It defies convention by incorporating practical research into the composition, color treatment, and texture effect of the image during the creative process. It attempts to learn from and integrate the schema in order to create modern works in a modern style and oil painting style [17].

### 2.3. Oil Painting Texture and Color Texture Feature Extraction and Principle

**Figure 4:**
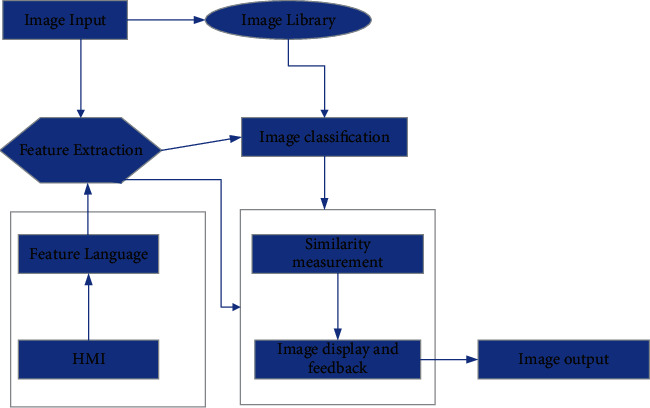
Flowchart of feature extraction.

In the previous article, we know the importance of texture and color texture to the image, so if we want to form the oil painting style, we need to extract the texture and color texture features of the classic oil paintings. The specific process of extraction is shown in Figure 4.

As shown in Figure 4, the feature extraction process first inputs the pictures into the gallery, then compares the similarity of the oil paintings in the gallery, extracts the places with low similarity, and then improves it.

For the extraction of color texture features in the image, the histogram method of RGB space is generally adopted. The RGB model is the most commonly used color representation method in the computer, and the statistical histogram of the image is a function, which is usually expressed as

(1) $$\begin{array}{c} G\left(v\right)=\frac{A_{v}}{m},\;v=0,1,2,\ldots ,N. \end{array}$$

**Figure 5:**
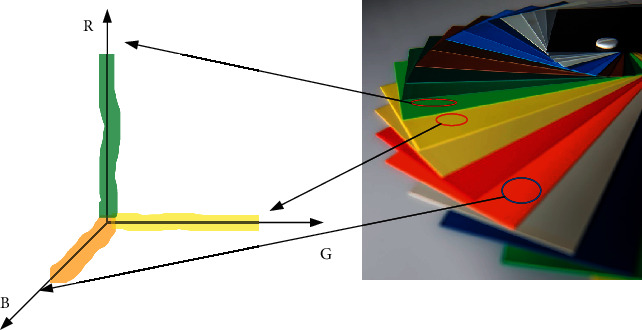
Statistical histogram.

In the upper form, *v* represents the color texture features that need to be extracted in the image, and *m* is the feature dimension of the feature in the RGB model. In order to make the size change of the picture not affect the image feature recognition, this paper normalizes the function. For the color of the image, the RGB model will obtain the statistical histogram of three different colors: R, G, and B, as shown in Figure 5.

If the histograms intersect, the horizontal axis is generally used to classify each color. The level depends on the depth of the color, and the vertical axis represents the proportion of the color in the Figure. If AG (*v*) and AW (*v*) are similar features of the two pictures, then the color similarity of the two images is calculated as follows: *G* and the characteristic histogram of the image *W* in the image library.

(2) $$\begin{array}{c} S\left(G,W\right)=\frac{\sum_{v=0}^{l-1}\min\left[A_{G}\left(v\right),A_{W}\left(v\right)\right]}{\sum_{v=0}^{l-1}H_{Q}\left(v\right)}. \end{array}$$

In the query matching system, the efficient and accurate similarity matching degree of the query system makes the two methods restrict each other. At the same time, in order to improve efficiency, it also needs to reduce the cost of calculating the same distance. The original search method based on color features filters out the pictures with completely different colors and then introduces the similarity with the texture features of the image. It realizes the search based on texture characteristics, which further improves the accuracy of the search [18]. The fastest matching method is the distance between objects of different colors in the same picture, which roughly represents the color features on the three axes of R, G, and B. Then, the image color features can be represented by vectors:

(3) $$\begin{array}{c} F=\left[\kappa_{R},\kappa_{G},\kappa_{B}\right]. \end{array}$$

At this time, the matching degree between image G and image W is as follows:

(4) $$\begin{array}{c} S\left(G,W\right)={\sqrt{\left(F_{G}-F_{W}\right)}}^{2},\\ \sqrt{{\left(F_{G}-F_{W}\right)}^{2}}=\sqrt{\sum_{R,G,B}{\left(\kappa_{G}-\kappa_{W}\right)}^{2}}. \end{array}$$

However, the RGB model histogram cannot effectively measure the fuzziness of people's perception of color features. In order to accurately measure the similarity of two different images, it is necessary to retrieve people's visual color similarity so as to integrate modern art creation into oil painting style. Therefore, it is necessary to refer to the color Table [19]. Assuming that people can feel the same reference color visually, and the number of this group of reference colors is more than the color features of the image to be retrieved, the matched feature vector in the cumulative histogram is

(5) $$\begin{array}{c} F={\left[e_{1},e_{2},\sigma ,e_{N}\right]}^{T}. \end{array}$$

Among them, *e*_1_ represents the frequency of the reference color in the image, and *N* is the dimension coefficient of the color reference Table, then the similarity matching degree between the test image *A* and the image *W* in the image library is

(6) $$\begin{array}{c} S\left(G,W\right)=\sqrt[{N}]{{\left(F_{G}-F_{W}\right)}^{2}},\\ \sqrt[{N}]{{\left(F_{G}-F_{W}\right)}^{2}}=\sqrt{\sum_{i=1}^{N}N_{1}{\left(e_{iG}-e_{iW}\right)}^{2}}. \end{array}$$

In modern art creation, we need to match the similarity between the newly created image and the color of classic oil painting in the gallery step by step. The closer the similarity is, the deeper the integration of color and texture in classical oil painting is, and the oil painting style is forming [20]. Of course, in order to form the more perfect oil painting style, it is necessary to extract and recognize the texture features in classical oil painting [21]. At present, the commonly used texture analysis methods are statistical analysis method and spectrum analysis method. The measurement of texture has four feature quantities, namely contrast, energy, entropy, and correlation [22].

The so-called contrast refers to the moment of inertia of the main diagonal. The calculation method is generally as follows:

(7) $$\begin{array}{c} CON=\sum_{G}\sum_{W}{\left(G-W\right)}^{2}d_{GW}. \end{array}$$

Texture is divided into thickness. For coarse texture, the value of *d*_*GW*_ is a value concentrated on the diagonal of the image. If the value of (*G*-*W*) is smaller, the value of image contrast is smaller, otherwise, it is larger. The calculation of energy will be affected by the gray value of the image, so assuming that the energy is *t*, the formula is

(8) $$\begin{array}{c} T={\sum_{G}\sum_{W}\left(d_{GW}\right)}^{2}. \end{array}$$

Because the gray value of the image will affect the recognition of the texture features of the image, it is necessary to measure the uniformity of the gray value distribution of the image. When *d*_*GW*_ is more concentrated on the diagonal of the image, the value of *T* will be larger, and vice versa. When the dispersion degree of *d*_*GW*_ value distribution in the gray level co-occurrence matrix is different, the entropy value will change. The more dispersed *d*_*GW*_ is, the greater the entropy value will be. The calculation formula is as follows:

(9) $$\begin{array}{c} C=-\sum_{G}\sum_{W}d_{GM}\log\;d_{GM}. \end{array}$$

The correlation is a measure of the linear relationship between image gray levels. Assuming that the mean value in matrix *d*_*GW*_ is *λ*_*a*_ and *λ*_*b*_, and the standard deviation is *χ*_*a*_ and *χ*_*b*_, the formula for calculating the correlation is

(10) $$\begin{array}{c} COR=\frac{\left[\sum_{G}\sum_{W}GWd_{GM}-\lambda_{a}\lambda_{b}\right]}{\chi_{a}\chi_{b}}. \end{array}$$

Therefore, as an example, features can be basically extracted, and then roughness is the measurement of texture granularity, fine or rough. The finer the texture, the finer the image, and the grain size is the size of the basic grain of the texture [23], which can be defined as

(11) $$\begin{array}{c} R=\left[\left(\frac{1}{X_{ps}}\sum_{l,v\in N_{t}}{\left[\vartheta \left(R,l,j\right)\right]}^{2}\right)>Y\right]. \end{array}$$

In the above formula, *ϑ*(*R*, *l*, *j*) represents the window with coordinates (*I*, *J*) as the center, *R* as the radius, Npt represents the clarity of the image, and *Y* is the threshold, so the image texture features can be extracted through granularity. Therefore, the extraction principles of color texture features and texture texture features are integrated into the depth neural network model. It integrates the color texture features and texture features of the image into the depth neural network model. Through the autonomous learning ability of the model, it no longer needs to extract step by step according to the above principle. It can not only improve the extraction efficiency of image features but also improve the accuracy of similarity and texture roughness. In the depth neural network model, the image contrast is defined as

(12) $$\begin{array}{c} CTS={\left(\frac{H}{a_{4}}\right)}^{1/4}. \end{array}$$

Among them, A4 =  *λ*_*a*_/*H*^4^, H is the standard deviation, and the calculation method is as follows:

(13) $$\begin{array}{c} H=\sqrt{\left(\lambda_{b}-\chi_{b}^{2}\right)}\\ =\sum_{i=0}^{U}p,\\ H_{4}=\sum_{i=0}^{U}p_{4}. \end{array}$$

In the upper form, *H* is the intensity of image brightness, and *P* represents the probability of texture appearing in the image with the same roughness. Therefore, in the deep neural network model, the classical oil painting is put into the neural network model, and then the modern works are transmitted to the deep neural network model for comparison. Then, the similarity between color texture and texture characteristics is obtained, and modern works are constantly adjusted to form oil painting style.

## 3. Experiment of Oil Painting Texture and Color Texture Feature Extraction

### 3.1. Experiment of Texture Feature Extraction and Recognition

**Figure 6:**
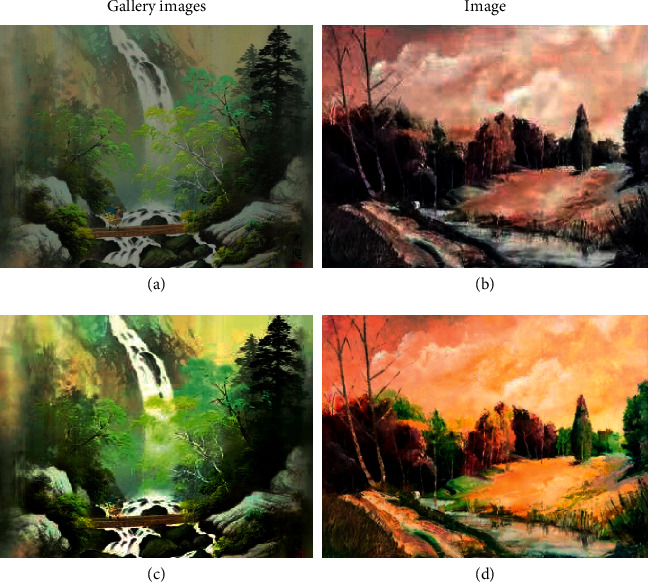
Experimental sample.

This experiment will take two pictures as samples and use the depth neural network model constructed in this paper to recognize the texture of the two pictures. One of the samples is a classic oil painting in the model, while the other is a work of modern art. The sample picture is shown in Figure 6.

The specific data of each picture in Figure 6 is shown in Table 1.

**Figure 7:**
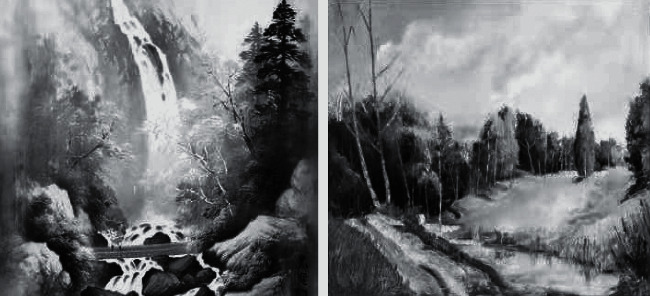
Sample picture texture.

Therefore, the texture of the picture is recognized and extracted in this experiment, and the texture is shown in Figure 7.

Figure 7 shows that the sample picture's texture is delicate, and it is clear that the painter is attempting to depict a peaceful landscape. The painter is very light when it comes to texture in the painting process. The image on the left is a classic gallery piece, while the image on the right is a contemporary art piece. Because the picture on the right cannot reflects the painter's emotion during the painting process, the similarity in texture should be around 85 percent. The image on the left is more delicate, particularly the texture of the waterfall, which will convey more vivid thoughts and feelings downstream.

### 3.2. Color Texture Feature Extraction Experiment

**Figure 8:**
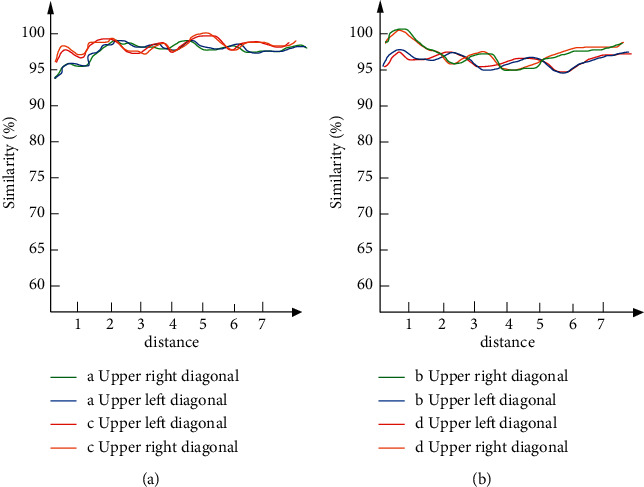
Diagonal color similarity. (a) Similarity between A and C. (b) Similarity between B and D.

Taking the pictures in the above experiment as samples, the above A, B, C, and D pictures have different brightness. Therefore, this experiment first identifies the color similarity of the same picture under different brightness. Taking the diagonal of the picture as the benchmark, the measurement data record of similarity is shown in Figure 8.

**Figure 9:**
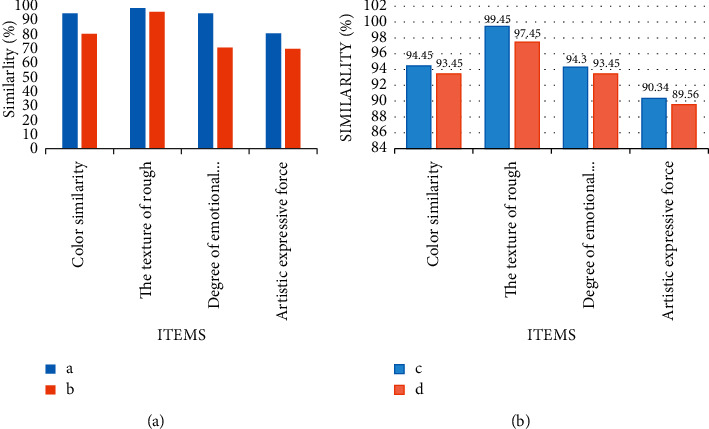
Similarity of different pictures. (a) Comparison of A and B. (b) Comparison of C and D.

In Figure 8, seven color points are tested from the diagonal of the picture, and the color similarity of the same picture under different brightness is identified. It can be found that the color similarity of the color taking points of the same diagonal in the picture is basically the same. In particular, although the same picture has different clarity, the color similarity is very similar and basically the same. Therefore, the model constructed in this paper has high accuracy for the extraction of color similarity, and it is of great help to the formation of oil painting style. Therefore, we extract and recognize the texture features and color features in the sample image, as shown in Figure 9.

In order to better measure the recognition of color features and texture features in this model, this experiment also makes similarity statistics on the color and texture features of the same picture of different pixels in the sample, as shown in Table 2.

In combination with Figure 9 and Table 2, the pixels of the image will affect the similarity of the image, so it is necessary to ensure the clarity of the image during image comparison. In addition, from Figure 9, there will be some gaps between the color and texture of classic oil paintings and modern art works. However, it can be constantly revised according to the gap to guide the final formation of oil painting style.

### 3.3. Experimental Summary

Through the experiment of this paper, the model constructed in this paper plays a very important role in the formation of oil painting style. The texture extraction of oil painting is in place, which can clearly see how the painter writes in the process of painting. This model can find out the gap between classical oil paintings and modern art works by comparing the color characteristics, texture, and texture characteristics. It can help painters approach the oil painting style purposefully and promote the formation of oil painting style in modern art creation. At the same time, the analysis of the texture and other characteristics of the image is the analysis of the schema, and the model in this paper has a good effect on the schema analysis.

## 4. Discussion

This paper understands the texture characteristics of oil painting. The texture of oil painting can clearly see the trend of painters' brushes in their creation. A careful study of the texture of classic paintings can be of great help to artistic creation. The artistic expression of oil painting is very strong, especially in the matching of colors. The collocation is very strong, and some have a great visual impact. In the painting materials of oil painting, the roughness of texture produced by the thickness of pigment is different. Therefore, some that need to be distinguished can be reflected by the thickness of the pigment. Therefore, the integration and development of oil painting style and modern art can promote the development of modern art. Schema is very important in portrait, and point, line, and surface lighting constitute the basic structure of portrait. In addition, illustration is essential to the art of painting, especially in the modeling and placement of things in the portrait. This reflects the overall artistic effect of the portrait, so the schema can also be carefully analyzed to promote the development of modern art. Through the experiment of this paper, it can extract and recognize the texture and color texture features in oil paintings, and analyze the schema composition in oil paintings. This model can further compare the gap between classical oil painting and modern art works, and automatically make modern works close to oil painting style and promote the formation of oil painting style. It can also make a very detailed analysis of the schema.

## 5. Conclusion

This paper explores the role of texture and color texture in the portrait. Texture is the most direct way for oil painting to convey the painter's thoughts and feelings, and it is also the best way to convey the artistic effect of portrait. Color texture can bring great visual impact to people. Therefore, this paper makes a theoretical reasoning on how to form oil painting style and graphic analysis. This paper also constructs a deep neural network model to facilitate the extraction of image texture and color texture features so as to facilitate the graphic analysis in modern creative art and the formation of oil painting style. And the experiment of this paper proves that through the combination of the recognition principle of image color and texture features and the depth neural network model, we can well compare the gap of modern art in oil painting style and promote the formation of oil painting style. However, the research on oil painting texture and color texture features in this paper will still be affected by many uncertain factors. It is hoped that future research can overcome the influence of uncertain factors.

## Figures and Tables

**Table 1:** Picture data.

Picture	Luminance (%)	Pixel
*a*	85	128 ∗ 138
*b*	80	192 ∗ 192
*c*	90	384 ∗ 384
*d*	95	640 ∗ 640

**Table 2:** Similarity of color and texture features of the same image with different pixels.

	Gallery images		Image	
	*a*	*c*	*b*	*d*
Color similarity	99.45	97.45	89.45	94.45
The texture of rough	94.5	94.5	84.45	95.67
Degree of emotional communication	93.23	98.34	93.45	96.45
Artistic expressive force	94.56	95.45	94.34	98.45

## Data Availability

The data used to support the findings of this study are available from the corresponding author upon request.
